# Nucleic acid-based approaches to investigate microbial-related cheese quality defects

**DOI:** 10.3389/fmicb.2013.00001

**Published:** 2013-01-21

**Authors:** Daniel J. O'Sullivan, Linda Giblin, Paul L. H. McSweeney, Jeremiah J. Sheehan, Paul D. Cotter

**Affiliations:** ^1^Food Bioscience Department, Teagasc Food Research CentreFermoy, Ireland; ^2^School of Food and Nutritional Sciences, University College CorkCork, Ireland; ^3^Alimentary Pharmabiotic Centre, University College CorkCork, Ireland

**Keywords:** molecular methods, cheese quality defects, microbial defects

## Abstract

The microbial profile of cheese is a primary determinant of cheese quality. Microorganisms can contribute to aroma and taste defects, form biogenic amines, cause gas and secondary fermentation defects, and can contribute to cheese pinking and mineral deposition issues. These defects may be as a result of seasonality and the variability in the composition of the milk supplied, variations in cheese processing parameters, as well as the nature and number of the non-starter microorganisms which come from the milk or other environmental sources. Such defects can be responsible for production and product recall costs and thus represent a significant economic burden for the dairy industry worldwide. Traditional non-molecular approaches are often considered biased and have inherently slow turnaround times. Molecular techniques can provide early and rapid detection of defects that result from the presence of specific spoilage microbes and, ultimately, assist in enhancing cheese quality and reducing costs. Here we review the DNA-based methods that are available to detect/quantify spoilage bacteria, and relevant metabolic pathways in cheeses and, in the process, highlight how these strategies can be employed to improve cheese quality and reduce the associated economic burden on cheese processors.

## Introduction

There are approximately 1000 varieties of cheeses, corresponding to nine different cheese families (Cheddar, Dutch, Swiss, Iberian, Italian, Balkan, Middle Eastern, Mould-ripened, and Smear-ripened) produced worldwide (Sandine and Elliker, 1970; Fox and McSweeney, 2004; Fox et al., 2004). Cheese is one of the most traded dairy products in the world with EU production of more than 8.4 million tons in 2011 (www.eurostat.eu). This generates huge revenues for leading cheese exporting economies. The primary ingredients of cheese are milk, rennet, and salt. However, it is microbial interactions with these major ingredients which allows for the production of the different varieties. These microbial populations are also the least controllable factor in cheese production (Fox, 2000; Jany and Barbier, 2008).

Microbial populations in cheese can be split into two distinct groups i.e., starter and non-starter microorganisms. Generally, starter and non-starter populations exhibit an inverse numerical relationship, with starter culture populations dominating during early cheese manufacture, but decreasing in number throughout the ripening process to be eventually replaced by the secondary microbiota. The starter microbiota cause rapid acidification via the production of lactic acid and produce enzymes that are important for flavor development during ripening (Leroy and De Vuyst, 2004). The most commonly used starter cultures are from the genera *Lactococcus, Lactobacillus, Streptococcus, Leuconostoc*, and *Enterococcus* (Beresford et al., 2001) and are used as either pure or mixed cultures (McSweeney, 2007). Non-starter/secondary organisms are primarily bacteria but can also include yeasts, molds, and filamentous fungi (Fox, 2000). Secondary, or initially subdominant microbiota, and in particular non-starter lactic acid bacteria (NSLABs), can play a key role in ripening and flavor development, for example, propionic acid bacteria (PAB) and/or smear cultures (including *Brevibacterium linens*). However, they can also be associated with the occurrence of defects. NSLAB are adventitious bacteria that gain access to cheese via the ingredients used and/or the production and ripening environment. They occur as heterogeneous populations with cell densities exceeding 10^6^ cfu/g cheese during the ripening process (Swearingen et al., 2001). They primarily consist of facultatively heterofermentative (mesophilic) lactobacilli (FHLs) as well as Pediococci, Enterococci, and Leuconostocs (Beresford et al., 2001; Beresford and Williams, 2004). FHLs are Gram-positive non-motile bacteria capable of growth at pH ranging from 5.5 to 6.2, in 4–6% salt and temperatures from 2°C to 54°C (Lynch et al., 1996). It is the relationship between these non-starter microbes and the physical features of the cheese (salt, pH, and moisture) that can lead to specific (un)desirable characteristics (Ndoye et al., 2011).

Defects caused by microorganisms that affect the quality of cheese include odor and taste defects, biogenic amine (BA) formation, gas formation, and secondary fermentations, mineral deposition and, potentially, cheese pinking. Controlling the strains, and the proportions thereof, is emerging as a key issue to minimize cheese defects (McSweeney, 2007).

There are a number of strategies which can be employed to facilitate the detection of microorganisms that cause defects. Traditional culture-dependent studies, although relatively inexpensive, suffer biases due to difficulties encountered when culturing many microbes present in the cheese matrix (Ndoye et al., 2011). Molecular methods, based on DNA and/or RNA isolation, provide alternative strategies. Some of the molecular approaches which have been quite popular, such as PCR-based denaturing gradient gel electrophoresis (DGGE), temporal temperature gradient electrophoresis (TTGE), single strand conformation polymorphism (SSCP), and terminal–restriction fragment length polymorphism (T-RFLP) techniques, are in turn being replaced by quantitative real time PCR (qRT-PCR) and next generation sequencing (NGS) technologies (Jany and Barbier, 2008; Ndoye et al., 2011). These methods are highly accurate and, in the former case, rapid, and cost effective. Furthermore, these approaches facilitate the detection of both specific microbial populations and of encoded metabolic pathways as the need arises (Mardis, 2011). This paper reviews molecular methods which are currently employed to detect spoilage bacteria in cheese matrices and discusses the potential use of NGS platforms for the cheese industry.

## Defects associated with cheese and the bacteria responsible

Defects can occur in cheese due to variations in milk quality, milk pre-treatment (pasteurization), hygiene practices, differences in starter culture activity and acidity profiles, manufacture technology, compositional parameters, and ripening temperature/environments. In addition, consumer demand has seen manufacturers endeavor to reduce the salt content of cheese. This in turn has resulted in a noticeable increase in the occurrence of cheese defects due to increased bacterial growth (McSweeney, 2007). Many defects are cheese type specific and a selection of defects is presented here that illustrate the influence of microbiota on cheese quality.

### Aroma and taste defects

The production of volatile flavor compounds by cheese microbiota is considered a crucial characteristic of cheese quality. However, when certain limits are exceeded, or where an imbalance of flavor compounds occurs, flavor defects are observed (McSweeney, 2007). Common taste/aroma defects caused by cheese microbiota include bitterness, hydrolytic rancidity, and sulphurous defects (Lemieux and Simard, 1991). Bitterness defects, common in Cheddar and Gouda as well as in low salt and low fat cheese, can be as a result of either excessive proteolysis of caseins or low bacterial peptidase activity among starters (Sousa et al., 2001). Bitter hydrophobic peptides can be liberated from the C-terminal region of β-casein and in α−_s1_-casein and are liberated through the activity of proteinases (Lemieux and Simard, 1991; McSweeney, 2007). These enzymes, produced by psychrotrophs, such as *Pseudomonas fluorescens* and *P. putrefaciens*, are heat stable and thus unaffected by pasteurization temperatures consequently allowing for bitter flavors to accumulate (Lemieux and Simard, 1991). Changing the coagulant, using a starter with high peptidase activity and/or manipulating salt content can reduce the occurrence of such bitterness (Lemieux and Simard, 1991).

Hydrolytic rancidity occurs as a result of lipolysis whereby lipids undergo hydrolytic degradation to free fatty acids (FFAs). Levels of FFAs are often used as indicators of lipolysis. Starter cultures, non-starter LAB, and moulds/smear organisms all produce lipases that cause lipolysis during ripening (McSweeney, 2007) and thus have the potential to cause hydrolytic rancidity. Most LAB have a low lipolytic ability and it is the number of bacteria and the time in contact with the cheese fat that leads to the production of significant levels of FFA (Collins et al., 2003). PAB are considerably more lipolytic than LAB. Moulds such as *Penicillium* spp. are also strong lipolytic agents and are used in mound ripened cheeses such as Brie and Camembert (Collins et al., 2003; McSweeney, 2007).

Volatile agents such as sulphur compounds including (di)/methyl sulphide play a key role in the flavor of many surface ripened and soft cheeses. These compounds give off sulphurous, over ripened and garlic like flavors that contribute to the characteristic flavors associated with surface ripened cheeses such as Brie, Camembert, and Limburger. Coryneform bacteria, and *B. linens* in particular, are known to be the major producers of sulphur compounds. Flavor thresholds of these compounds are low (9–170 ppb for dimethyl sulphide in Camembert) and thus if these limits are exceeded cheese flavor is adversely affected (Sable and Cottenceau, 1999).

Other microbe associated flavor defects include fruity off flavors, harsh, and green flavors. Fruity off flavors are as a result of production of ethyl esters by some species of *L. lactis* and *L. lactis* subsp. *cremoris*. This can be controlled by careful selection of starter cultures capable of producing the correct flavor associated with a cheese type as well as standardizing storage/handling practices (Sandine et al., 1972; Sable and Cottenceau, 1999). Methyl alcohols/aldehydes produced by certain strains of *L. lactis* are also associated with off flavors (Morales et al., 2003). Harsh and green flavors are often caused by excessive production of acetaldehyde by some strains of *L. lactis* subsp. *cremoris.* This can be controlled through careful starter culture selection, particularly those high in aldehyde reductase, and the inclusion of *Leuconostoc* populations (Sandine et al., 1972). Leuconostoc species are known to antagonize detrimental bacteria through formation of organic acids and bacteriocin production (Daba et al., 1991; Stiles, 1994).

### Gas defects: split defects and secondary fermentations

Gas defects in cheese can occur for a variety of different reasons. Excess gas production in cheese manifests as cracks, slits, holes, and eyes, which while not harmful to the consumer, affects aesthetic properties (Sheehan, 2011). A variety of microbes can be responsible for gas defects. Gas defects can be subcategorized as either early or late gas. Early gas occurs within 1–2 days of manufacture and can affect many cheese varieties. Late gas occurs during later stages of ripening and primarily affects Dutch and Swiss-type cheeses (Jimeno et al., 1995; Mullan, 2000; McSweeney, 2007; Sheehan, 2011).

#### Early gas production

Poor hygiene or the use of unpasteurized milk can result in the presence of coliforms such as *Enterobacter, Escherichia, Citrobacter*, and *Serratia* which are strongly associated with early gas defects. These microbes produce H_2_ and/or CO_2_ gas aerobically or anaerobically as a by-product of lactose utilization (Sheehan, 2011). H_2_ is poorly soluble in the aqueous phase of curd and therefore even small quantities can cause serious gas problems. The presence of these gases often also results in development of off-flavors. Coliform levels of approximately 10^7^ cfu/g of cheese are sufficient to cause early gas defects (Mullan, 2000; Sheehan, 2011). Starter bacteria, including sub-species of *L. lactis, Streptococcus*, and *Leuconostoc*, have also been implicated in undesirable early gas production. Both *Lactococcus* and *Leuconostoc* species are capable of fermenting lactose and citrate to form CO_2_. Early gas formation problems often arise when the proportions of these starter bacteria differ from normal allowing one or a group of bacteria to predominate over others (Mullan, 2000; Sheehan et al., 2008). Yeasts such as *Kluyveromyces, Debaryomyces*, and *Candida* are also known to cause gas blowing issues in hard, semi-hard and soft cheeses. Such yeasts are highly resistant to commercial cleaning practices (McSweeney, 2007; Sheehan et al., 2008; Sheehan, 2011).

#### Late gas production

In many instances this phenomenon is due to the action of PAB which ferment lactose and/or lactate to propionic acid. This gives the characteristic “nutty” taste and results in the presence of the characteristic “eyes” associated with Swiss type cheeses (Sheehan et al., 2008). In these cases selected strains of PAB are purposely added along with the starter culture to produce different flavor profiles. However, in raw milk cheeses, such as Beaufort, the presence of PAB in milk leads to spontaneous, uncontrolled fermentations (McSweeney, 2007).

Late gas defects in Swiss and other cheese types can occur within a few weeks of manufacture and up to 4–6 months into ripening. There are several factors attributed to irregular late gas production including the presence of butyric acid bacteria (*Clostridium* spp.), FHLs, salt tolerant Lactobacilli, and the abnormal growth of PAB (White et al., 2003; McSweeney, 2007; Sheehan et al., 2008; Daly et al., 2010; Sheehan, 2011). Butyric acid bacteria are anaerobic bacteria that ferment lactate to butyric acid, CO_2_, and H_2_. These gases are produced when *Clostridium tyrobutyricum* spores germinate during cheese ripening. Other butyric acid bacteria species known to contribute to late gas defects via spore germination include *C. butyricum, C. sporogenes*, and *C. beijerinckii* (Sheehan, 2011). Swiss cheese, and Emmental in particular, is particularly susceptible to spore germination due to the anaerobic environment of cheese as well as higher ripening temperatures (in excess of 20°C). The low salt and acid content also assists in spore germination. Spores often enter milk via fecal contamination of cows udders and are capable of surviving high temperature pasteurization (Sheehan, 2011). Good hygiene practices, with respect to both milk and manufacturing equipment, combined with microfiltration or bactofugation of cheese milk reduces the possibility of contamination. Enzymes added to the cheese milk such as lysozyme and the use of bacteriocins such as nisin may also be used in preventing contamination with clostridia spores. Nitrates are also often added for preservation purposes (McSweeney, 2007; Sheehan, 2011).

FHLs, salt tolerant and mesophilic lactobacilli cause gas blowing in Cheddar-type and brine salted cheeses (Sheehan, 2011). FHLs such as *L. brevis* ferment residual lactose to CO_2_ during ripening. This issue is more pronounced in raw milk cheeses due to high levels of NSLAB in comparison to cheese made from pasteurized milk (Sheehan, 2011). *L. brevis* is also present in pasteurized milk but at lower levels due to pasteurization and competition by other NSLABs such as *L. paracasei* (Daly et al., 2010). Salt tolerant and mesophilic lactobacilli have been implicated in irregular gas production in both Swiss and Dutch type cheeses. Rapidly growing starter bacteria limit the amounts of lactose and galactose present in the cheese and consequently less is available for NSLAB growth (Daly et al., 2010; Sheehan, 2011). PAB, and *P. freudenreichii* in particular, are responsible for regular eye formation in Swiss-type cheese. However, abnormal growth can lead to late gas defects occurring. Different sub-species of *P. freudenreichii* can have different effects on flavor profile and eye formation. Research has shown that the PAB strains selected, as well as co-cultivation strains, such as *L. helveticus* which produces peptides that stimulate activity of PAB particularly during cold room storage, can have a dramatic effect on the occurrence of split defects (White et al., 2003). For example, PAB strains with high aspartase activity are associated with excess gas formation. Aspartase is an enzyme responsible for the deamination of aspartate and varies in activity among different strains of PAB. Lactate, in the presence of aspartate, is fermented to acetate, succinate and CO_2_ by PAB. Therefore, the presence of strains with high aspartase activity causes excess secondary fermentation (Daly et al., 2010).

### BA formation

**Figure 1:**
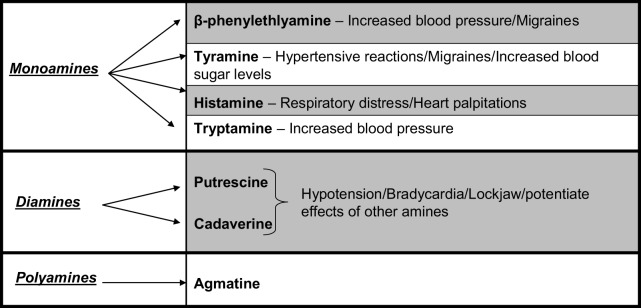
**Food associated amines and their effects**.

BAs are aliphatic, heterocyclic or aromatic organic nitrogenous compounds with low molecular weight that can be found in a variety of foods including cheese, fish, wine, beer, and dry sausage (Shalaby, 1996; Bover-Cid and Holzapfel, 1999; Novella-Rodriguez et al., 2003; Önal, 2007). They are also naturally present in the body where they function as neurotransmitters and signal transducers (Santos, 1996). BAs can be further sub-divided into monoamines, such as tyramine, and polyamines, such as putrescine, agmatine, and spermidine (Bover-Cid and Holzapfel, 1999; Novella-Rodriguez et al., 2003). These amines can exhibit a toxic effect, with reports that histamine concentrations as low as 20 mg/kg cheese can elicit an adverse reaction in some humans (Shalaby, 1996; Novella-Rodriguez et al., 2003). They affect both the vascular and nervous systems (Spano et al., 2010), with ingestion in susceptible individuals causing a diverse range of symptoms including headache, cardiac palpitations, localized inflammation, nausea, vomiting, and hyper/hypotension (Shalaby, 1996) (Figure 1). BAs have been associated with cases of food poisoning, particularly in fish and cheese, hence the terms scombroid fish poisoning and “the cheese reaction” have been coined (Joosten and Northolt, 1989). Individuals that are susceptible to adverse reactions following BA ingestion include those prescribed antidepressant drugs classed as monoamine oxidase inhibitor drugs (Santos, 1996; Shalaby, 1996; Bover-Cid and Holzapfel, 1999; Innocente et al., 2007; Spano et al., 2010) or those with an impaired detoxification system. Furthermore, biogenic amines are also known precursors of carcinogens (Joosten, 1988; Joosten and Northolt, 1989; Giraffa, 2003).

The bacteria responsible for the production of biogenic amines contain an amino acid decarboxylase which removes the α-carboxyl from a particular amino acid to give the corresponding amine. BAs and corresponding amino acids include: histamine (histidine), tyramine (tyrosine), tryptamine (tryptophan), putrescine (ornithine), cadaverine (lysine), and β-phenylethylamine (phenylalanine) (Shalaby, 1996). Several species of *Lactobacillus, Clostridium, Pseudomonas* as well as *Enterobacteriaceae* display decarboxylase activity (Shalaby, 1996; Giraffa, 2003). While most BAs are produced via decarboxylase activity, amines such as putrescine are produced by LAB, of the genera *Enterococcus* and *Lactobacillus*, through deamination of agmatine by agmatine deaminase (Ladero et al., 2012). In cheese, biogenic amines are generally produced by the non-starter microorganisms (Stratton et al., 1991; Shalaby, 1996; Spano et al., 2010). Non-starter microbiota capable of BA formation includes *L. bulgaricus, L. buchneri, L. curvatus, L. casei, L. acidophilus Enterobacter, Escherichia, Citrobacte*r, and *Klebsiella*. Certain PAB species have also been implicated in BA formation (Joosten, 1988; Joosten and Northolt, 1989; Sumner et al., 1990; Shalaby, 1996; Marino et al., 2000; Spano et al., 2010). It is also noteworthy that certain strains of starter microbiota such as *L. lactis* and *L. helveticus* are capable of BA formation, although this has become less of an issue due to screening for decarboxylase activity (McSweeney, 2007).

### Mineral deposition defects

Mineral deposition, corresponding to calcium lactate crystal (CLC) formation, is a common defect found in Cheddar cheese (Chou et al., 2003; Swearingen et al., 2004). CLCs appear as white crystals or spots on the external surface of the cheese (Johnson et al., 1990; Chou et al., 2003; Agarwal et al., 2005, 2006). While not harmful, CLC formation is often mistaken for mound by consumers. This results in an increase in complaints to the manufacturer often leading to product recall. CLC formation is influenced by the concentrations of calcium and lactate ions present in the cheese (Johnson et al., 1990; Chou et al., 2003; Swearingen et al., 2004). CLCs are formed via the racemization of L(+)-lactate to the less soluble D(−)-lactate by racemase-positive NSLAB. Agarwal et al., found that CLC crystals occurred after 56 days of ripening on cheese inoculated with *L. curvatus*, but not in *L. curvatus* negative cheese (Agarwal et al., 2006). Somers et al., provided further evidence to the role of *L. curvatus* in CLC formation by demonstrating that *L. curvatus* are capable of forming biofilms which survive cleaning practices. These biofilms can then detach from cheese vats and contaminate the cheese matrix (Somers et al., 2001; Swearingen et al., 2004; Agarwal et al., 2005). Other researchers have shown that many other strains of lactobacilli and Pediococci may also be involved in CLC formation (Swearingen et al., 2004). Chou et al., showed that lactobacilli negative cheese did not suffer from CLC formation. Furthermore, control cheeses and cheeses manufactured with *L. helveticus* did not suffer from crystal formation This study also suggests that accelerated maturation at higher than normal temperatures may accelerate NSLAB growth, and consequently D(−)-lactate formation and CLCs (Chou et al., 2003). Johnson et al., showed that CLCs did not form in cheeses that were gas flushed and vacuum packed. However, controlling populations of racemase positive lactobacilli and concentrations of lactic acid are regarded as more effective methods of controlling CLC formation (Johnson et al., 1990).

### Cheese pinking

Pink discoloration defects can occur either on external surfaces or within the cheese matrix (Shannon et al., 1969; McSweeney, 2007). This defect may occur in cheese with or without Annatto. Annatto is a carotenoid food dye comprised mainly of two pigments (bixin and norbixin), sourced from the seeds of the Achiote tree. This dye, which gives an orange/red color to cheese, often suffers from pink discoloration due to photo-oxidation of its pigments, or interactions of the pigments with heat and/or light (Shumaker and Wendorff, 1998). However, natural non-dyed cheeses can also suffer from pinking. In such cases, thermophilic lactobacilli (particularly *L. delbruechii* subsp. *Bulgaricus*, and *L. helveticus*) and PAB (*P. shermanii*) have been suggested as potential causes, but this remains a matter of much debate (Shannon et al., 1969; McSweeney, 2007). Pink discoloration is not harmful to consumers but may result in product recall or downgrading (Shumaker and Wendorff, 1998).

## Detection methods

Molecular techniques have revolutionized the strategies employed to detect beneficial and detrimental microorganisms in foods. Previously, culture-dependent approaches, which relied on the isolation and cultivation of microbes, were exclusively employed. In these instances, the cultured microbes were identified based on their morphology and/or biochemical features (Jany and Barbier, 2008; Ndoye et al., 2011; Quigley et al., 2011). Although relatively inexpensive, such approaches are inefficient, time consuming and tedious. Furthermore, many bacterial species cannot be cultured easily, or at all, on standard agar plates. Thus, the identification and quantification of bacteria in this way is inherently biased toward those bacteria that grow well in a laboratory setting (Temmerman et al., 2004; Delbes et al., 2007; Ndoye et al., 2011). Selective media such as MRS, MSE, LM17, and KAA are widely used for culturing *Lactobacilli, Leuconostoc, Streptococci/Lactococci*, and *Enterococci*, respectively, from cheese (Rea et al., 2004; Landete et al., 2008). These media allow for the selection of the particular species in question only. In the past, BA producing species were detected in cheese by culturing on selective media containing a pH indicator, such as bromocresol purple. A color change is then noted around decarboxylase producing colonies due to the production of alkaline amines (Joosten and Northolt, 1989; Bover-Cid and Holzapfel, 1999; Landete et al., 2008; Spano et al., 2010). Examples include MRS-decarboxylase broth used by Rea et al., for determining production of biogenic amines by enterococci (Rea et al., 2004).

As an alternative to traditional culturing, molecular methods provide rapid, reproducible, accurate, and non-biased strategies to analyse microbial communities. These techniques allow for specific species identification in foods without the need to culture. Detection of both viable and non-viable bacterial cells, damaged or completely lysed cells is also possible (Sheehan et al., 2009). Furthermore, molecular techniques can be employed to search for particular enzyme-encoding genes such as amino acid decarboxylases. Identifying microbes that cause defects early in the cheese making process enables manufacturers to uncover and remedy potential sources of contamination quickly and thus minimize the risk of a product recall (Delbes et al., 2007; Carraro et al., 2011).

PCR amplification of a specific target sequence is often the key element with respect to molecular approaches to bacterial identification (Jany and Barbier, 2008). Frequently the target region within bacterial genomes is the 16S rRNA gene or the 16S/23S spacer region, either using species/genera specific primers or universal primers (Clarridge, 2004; Randazzo et al., 2009; Ndoye et al., 2011). The 16S rRNA gene is ubiquitous among bacteria, present at high copy number and there is an abundance of species-specific sequence information available in public databases (Scheu et al., 1998; Justé et al., 2008; Quigley et al., 2011). The 16S rRNA gene consists of highly conserved and highly variable regions making it ideal for bacterial typing (Temmerman et al., 2004; Ndoye et al., 2011; Quigley et al., 2011). Amplifying other conserved target genes that contain conserved and variable domains, such as those encoding the RNA polymerase subunit B (*rpoB*), phenylalanyl-tRNA synthase (*pheS*), elongation factor Tu (*tuf*), DNA repair gene (*recA*) or heat shock protein (*hsp60*), or alternatively, genes that are genera, species and strain specific, can also be very informative. As more and more sequencing information becomes publicly available, this targeted approach is becoming more popular (Justé et al., 2008; Randazzo et al., 2009; Ndoye et al., 2011).

The first step in amplifying bacterial genes involves extracting high quality DNA or RNA from a food matrix. This is often accomplished using mechanical homogenization in a salt based solution followed by lytic enzyme treatment (lysozyme, mutanolysin, proteinase K). Nucleic acids are then extracted by either phenol chlorophorm or spin column purification systems which use detergents such as guanidine thiocyanate (Duthoit et al., 2003; Ercolini, 2004; Parayre et al., 2007; Jany and Barbier, 2008; Falentin et al., 2010). RNA isolation is achieved in a similar fashion with care taken to remove ribonucleases which degrade this single stranded nucleic acid (Ulve et al., 2008). Extracted nucleic acid, or cDNA generated from RNA, then provides the template for PCR amplification using universal, species specific or gene specific primers, depending on the goal of the study, to generate PCR amplicons. Resultant PCR amplicons will vary in size and/or sequence depending on their bacterial origin (Randazzo et al., 2009).

There are, however, issues associated with PCR amplification that can affect the accuracy and reproducibility of the detection methods. The quality of the DNA extracted from the cheese source is the first barrier. The cheese matrix contains many PCR inhibitors such as salts, fats, and carbohydrates which need to be removed during the extraction procedure (Justé et al., 2008). The choice of PCR primers also influences the effectiveness of PCR as dominant and sub-dominant bacterial populations may not be amplified in a proportional manner and, furthermore, different species may differ in gene copy numbers (Justé et al., 2008). Preferential or differential PCR amplification may also lead to the introduction of a biased view of the community present (Reysenbach et al., 1992; Jany and Barbier, 2008). Preferential amplification of certain PCR templates can occur as a result of differences in GC contents and/or primer mismatches at template annealing sites (Walsh et al., 1992). Another issue affecting PCR is the formation of artefacts such as chimeric amplicons which can occur due to heteroduplex formation (Jany and Barbier, 2008). These issues can be overcome by including co-solvents, hot-starting DNA, or by using low numbers of PCR cycles (Reysenbach et al., 1992). It should also be noted that the amplification of DNA from dead cells may result in false positives. In order to overcome this RNA can be isolated and subsequently used to generate a cDNA template. Inhibitors such as ethidium bromide monoazide (EMA) or propidium monoazide (PMA) can be used to bind to and inactivate DNA from dead cells (Nocker and Camper, 2009).

### Molecular approaches to study cheese defects

**Figure 2:**
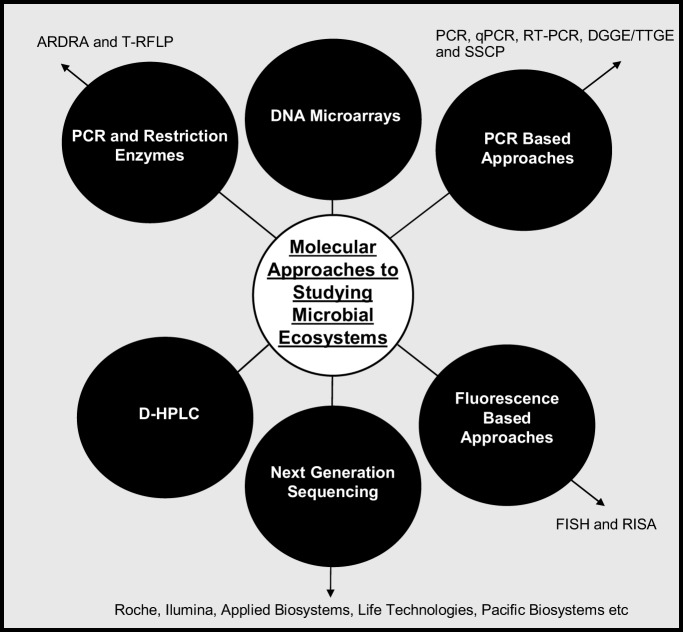
**Methods of profiling microbial ecosystems**.

Molecular techniques have not specifically been used to identify cheese defects but they have been used to profile microbial populations in cheese (Figure 2). Table 1 summarizes the techniques used, organisms identified and cheese tested.

**Table 1:** **Non-exhaustive list of genotyping methods to study microbiota of cheese and milk**.

**Authors**	**Method**	**Substrate**	**Type of study: microorganisms detected/genes targeted**
Rossi et al., 1999	Conventional Nested PCR	Raw Milk	Propionibacteria (*P. freudenreichii, P. jensenii, P. acidipropionici*)
Herman et al., 1997	Conventional PCR	Hard/Semi hard cheeses	*C. tyrobutyricum*
Ladero et al., 2008	qPCR	French/Spanish Commercial Cheeses	*hdc* gene
Fernández et al., 2006	qPCR	Milk, Cabrales Cheese	*hdcA G*ene
Ladero et al., 2010	qPCR	Raw/Pasteurized Milk	*tdcA* Gene
Lopez-Enriquez et al., 2007	qPCR	Innoculated raw and pasteurized milk cheeses	*fla* gene of *C. tyrobutyricum*
Falentin et al., 2010	qPCR and RT-PCR	Emmental Cheese	*P. freudenreichii* and *L. paracasei*
Graber et al., 2007	qPCR	Bovine milk cheese	*Staphylococcus aureus*
Hagi et al., 2010	qPCR	Raw milk cheese	*Listeria monocytogenes*
Cocolin et al., 2004	PCR-DGGE	Grana Padano cheese	*Clostridium* species
Randazzo et al., 2002	PCR-DGGE	Ragusano Cheese	*Lactobacillus* species
Randazzo et al., 2006	PCR-DGGE	Pecorino Siciliano cheese	Microbial biodiversity studies
Alegría et al., 2009	PCR-DGGE	Casín cheese	Lactic Acid Bacteria profiles
Giannino et al., 2009	PCR-DGGE	Fontina cheese	Microbial biodiversity studies
Bonetta et al., 2008	PCR-DGGE	Robiola di Roccaverno cheese	Microbial biodiversity studies
Florez and Mayo, 2006	PCR-DGGE	Cabrales cheese	Microbial diversity and succession
Alegria et al., 2012	PCR-DGGE	Oscypek cheese	Microbial biodiversity studies
Barbieri et al., 2012	PCR-DGGE	Fossa cheese	NSLAB biodiversity
Ogier et al., 2002	PCR-TTGE	Washed curd cheese	Differentiation between dominant microbes
Abriouel et al., 2008	PCR-TTGE	Alberquilla	LAB identification
Duthoit et al., 2003	SSCP	Salers cheese	Profile community dynamics
Saubusse et al., 2007	SSCP	Raw milk cheese	*L. monocytogenes* inhibition
Ercolini et al., 2003	FISH	Stilton cheese	Microbe visualization studies
Bunthof et al., 2001	FISH	Bovine milk cheese	LAB viability studies
Rademaker et al., 2005	T-RFLP	Tilsit cheese	Microbial dynamics studies
Cardinale et al., 2004	RISA	Goats milk	Microbial biodiversity studies
Ercolini et al., 2008	D-HPLC	Caciocavallo Silano cheese	Whey culture profiles
Treimo et al., 2006	DNA Microarray	Liquid cheese model	*Lactococcus* and *Propionibacteria* studies
Quigley et al., 2012	Roche Pyrosequencing	Artisanal cheeses	Microbial community analysis
Masoud et al., 2011	Roche Pyrosequencing	Danish raw milk and cheese	Microbial dynamics studies
Alegria et al., 2012	Roche Pyrosequencing	Oscypek cheese	Microbial biodiversity studies

#### Conventional and quantitative real time PCR (qRT-PCR)

Conventional PCR assays using genus- or species-specific primers to target 16S rRNA, or other genes commonly used for rapid bacterial detection and as such there are numerous publications on this topic (Hill and Wachsmuth, 1996; Lantz et al., 2000; Malorny et al., 2003). We will provide only two examples. Rossi et al., used nested PCR with species-specific primers to amplify propionibacteria from raw milk samples. This approach indicated seasonal variations in propionibacteria in the dairy environment (Rossi et al., 1999). Herman et al., used a similar approach to detect *C. tyrobutyricum* in hard and semi hard cheeses (Herman et al., 1997). Although such assays are useful from a detection perspective, they do not provide a very accurate insight into the quantity of the microbe present. In contrast, qRT-PCR quantifies the number of specific microorganisms or gene copies present in a sample and represents the “gold standard” in quantifying genes and gene expression (Bustin, 2005; Bustin et al., 2005; Postollec et al., 2011b). qPCR is rapid, extremely sensitive and has been applied in food microbiology, genomics, medicine, and environmental studies (Bustin et al., 2005; Quigley et al., 2011).

qRT-PCR differs from conventional PCR by virtue of being performed in real time in the presence of fluorescent reporters, such that the number of newly generated PCR amplicons can be quantified after each amplification cycle. A DNA binding cyanine dye, such as SYBR Green or BOXTO, are added to the reaction mixture and fluoresce during DNA amplification (Kubista et al., 2006). Probe based PCR is an alternative to fluorescent reporters.

Once one or many of the PCR components are depleted (primers, dNTP's, polymerase), a saturation limit is reached and the reaction stops. Fluorescence is then recorded vs. the number of cycles needed to reach saturation and from this the cycle threshold (Ct) is calculated (Bustin, 2005; Kubista et al., 2006; Postollec et al., 2011b). There are two methods by which the PCR product can be quantified i.e., absolute or relative quantification. Absolute quantification relies on a comparison between levels of fluorescence of the target amplicon to that of a standard curve of known amounts of the target amplicon. Relative quantification is based on gene expression vs. that of a “housekeeping gene,” a gene that is expressed at ubiquitous levels within the cell (Postollec et al., 2011a).

A recent review by Postollec et al., compiled numerous examples in which qRT-PCR has been applied to assess food safety (Postollec et al., 2011a). There are also cases in which qRT-PCR has been used to investigate food quality, and examples relevant to cheese are mentioned below. Decarboxylase and agmatine deaminase genes have been targeted by qPCR methods as part of efforts to detect, and ultimately target, biogenic amine producing bacteria (Ladero et al., 2008). Ladero et al., used a qPCR approach to detect histamine producing strains in 80 French and Spanish commercial cheese samples using histidine decarboxylase (*hdc*) specific primers. This approach allowed for *hdc* positive strains to be detected and quantified in cheeses long before the BAs could be detected via HPLC (Ladero et al., 2008). Fernandez et al., also developed a qPCR approach using *hdcA* specific primers for detecting histamine positive, Gram positive bacteria in both milk and cheese. Similarly, the blue cheese Cabrales, which has an inherently high BA content, was analyzed by qPCR as well as HPLC. Quantitative PCR detected *hdcA* positive bacteria in all samples during early ripening whereas histamine was only detected by HPLC on day 7 of ripening, even in the cheeses with the highest amine concentrations. Thus, while HPLC can detect actual levels of histamine (mg/g) in the final product, qPCR can determine if the bacteria responsible for histamine biosynthesis are present and in what numbers (Fernández et al., 2006). In another publication by Ladero et al., a qPCR method specific for the LAB tyrosine decarboxylase (*tdcA*) gene was used to detect and quantify tyramine producing bacteria in 57 raw or pasteurized cheese samples. *tdcA*-positive bacteria were found in all cheeses, in varying amounts, but the amine itself was only detected by HPLC in 56% of samples. This study implies that when tyramine producing bacteria exceed 10^4^ cfu/g cheese, as revealed by qPCR, tyramine build up becomes a quality/safety issue (Ladero et al., 2010). Further studies have also targeted putrescine decarboxylase genes. Strains of *Enterococcus, Lactococcus*, and *Lactobacillus* are all implicated in putrescine formation due to the presence of the agmatine deaminase gene cluster (AGDIc). A multiplex qPCR approach to detect and quantify the intergenic spacer region between *aguD* and *aguA* of the AGDIc was proposed by Ladero et al. In this study 29 cheese samples made from raw and pasteurized milk were analyzed for putrescine producers. Results determined that producers corresponding to the three genera were present in all except three cheeses. A direct correlation was also observed between cheeses with the highest numbers of putrescine producers and cheeses with the highest levels of putrescine present, as determined by HPLC. As with other qRT-PCR approaches, this method has the potential to facilitate the early detection of putrescine producers and/or levels of the deaminase gene in raw materials with a view to controlling putrescine levels in the final product (Ladero et al., 2012).

Late blowing issues caused by *C. tyrobutyricum* are a common problem in hard and semi-hard cheeses. As few as 50 spores per liter of milk is enough to cause late blowing effects and thus detection methods must be highly sensitive. Lopez-Enriquez et al., targeted the flagellin gene (*fla*) of *C. tyrobutyricum* to successfully detect spores in inoculated raw milk samples. Enzymatic treatment of samples prior to analyses allowed for detection of as few as 25 spores per 25 ml of raw milk (Lopez-Enriquez et al., 2007). Falentin et al. (2010) also performed studies using both qPCR and RT-PCR, the latter being employed to reflect RNA levels and thus metabolically active cells, to quantify levels of growth of *P. freudenreichii* and *L. paracasei* at different ripening stages of Emmental cheese. Monitoring these bacteria over time allows for greater understanding of LAB and PAB behavior in a complex cheese matrix and the roles they play in the occurrence of cheese defects (Falentin et al., 2010). Both *Staphylococcus aureus* and *Listeria monocytogenes* have also successfully been quantified in bovine milk and raw milk cheeses, respectively, using a qRT-PCR approach (Graber et al., 2007; Hagi et al., 2010).

Ultimately, it is conceivable that qPCR could be applied to any cheese defect associated with bacteria provided that there is sufficient genome sequencing data available to design target specific primers. Some issues may arise due to detection of dead cells but this can be overcome using inhibitors such as EMA and PMA. qPCR can therefore become a key tool in detecting and quantifying microorganisms known to contribute to cheese defects. Early detection prior to observation of a defect in the final product will enhance cheese quality and decrease overall costs.

#### Denaturing gradient gel electrophoresis (DGGE) and temporal temperature gel electrophoresis (TTGE)

DGGE/TTGE methods allow for separation of PCR amplicons based on differing sequences. These are among the most commonly used methods to assess complexity of microbial communities in food products (Ercolini, 2004; Bonetta et al., 2008) but are more typically employed for scientific rather than industrial applications. DGGE uses denaturing (urea and formamide-containing) acrylamide gels. As amplicons migrate through the gel matrix, the denaturing agents cause the amplicons to denature partially at melting domains within their sequence. Amplicons are separated due to differences in melting domains as a direct result of sequence differences (Ercolini et al., 2001; Ercolini, 2004; Cocolin et al., 2007; Jany and Barbier, 2008). DGGE is usually performed at a constant temperature between 55°C and 65°C (Ercolini et al., 2001; Ercolini, 2004). TTGE separates amplicons in the absence of denaturing chemicals and uses temperature variation over time to denature and separate DNA (Jany and Barbier, 2008). The addition of a GC clamp, a 30–40 bp GC rich region, added to the PCR primers ensures that amplicons do not completely degrade (Ercolini, 2004; Temmerman et al., 2004; Cocolin et al., 2007; Ogier and Serror, 2008). This approach yields banding patterns which reflect the complexity of microbial populations.

There are many examples where these technologies have been applied for identifying microbes in cheese, although, in the majority of cases, the detection of microorganisms responsible for cheese defects has not been a priority. Cocolin et al., optimized a protocol for using PCR-DGGE for directly detecting *Clostridium* species responsible for late blowing in cheese. Results obtained showed there was a strong correlation between DGGE and conventional plating techniques. This method has an estimated sensitivity of 10^4^ cfu/g cheese making it ideal for detecting spoiled samples (Cocolin et al., 2004). PCR-DGGE has also been used for investigating microbial biodiversity in artisanal and protected designation of origin (PDO) cheeses. Randazzo et al. (2002) and (2006), applied this method to Ragusano and Pecorino Siciliano cheeses, respectively. In the former case, *Lactobacillus* specific 16S rRNA primers were used to profile microbial communities. The biodiversity of cheeses provided from three different farmers was assessed and revealed the changes in microbial populations during the production process i.e., from raw milk, curd and 15–30 days old cheeses (Randazzo et al., 2002). The dynamics of the PDO cheese Pecorino Siciliano made from raw milk, raw milk plus starter culture, and pasteurized milk was investigated using a combined PCR-DGGE and culturing approach. Similar microbial profiles were observed in all three cheese samples, however, a predominance of wild *L. lactis* and *S. bovis* species in the raw milk cheese is likely responsible for the unique flavor associated with this cheese (Randazzo et al., 2006). The microbial composition of the Spanish artisanal cheese Casín, thought to be among the oldest traditional cheeses in Spain, was also investigated using both DGGE and standard culturing methods. Although the aim was to attempt to identify LAB to replace or complement those currently used, the results demonstrate the success of the technique for microbial detection. Interestingly, *S. thermophilus*, a species not previously isolated from traditional Spanish cheeses, was identified by PCR-DGGE but not by culturing methods. Added to this high numbers of coliforms, indicating poor hygienic practices, were identified in the initial stages of production but not in the final product sampled at day 30 (Alegría et al., 2009). Many other studies are available in which microbial populations in artisanal cheeses have been analysed using PCR-DGGE. These include, Fontina (Giannino et al., 2009), Robiola di Roccaverno (Bonetta et al., 2008), Cabrales (Florez and Mayo, 2006), Oscypek (Alegria et al., 2012), Fossa (Barbieri et al., 2012), and other raw milk cheeses (Quigley et al., 2011) (Florez and Mayo, 2006; Bonetta et al., 2008; Alegría et al., 2009; Giannino et al., 2009; Quigley et al., 2011; Alegria et al., 2012; Barbieri et al., 2012).

Ogier et al., applied a TTGE approach to investigate the microbiota of model miniature cheeses. This 16S approach was able to differentiate between dominant species such as *L. delbueckii* subsp. *bulgaricus, L. acidophilus*, and *L. delbrueckii* subsp. *lactis* within a cheese matrix. However, it failed to identify minor species that were present at concentrations below 1% making it unsuitable for the detection of many potential pathogens (Ogier et al., 2002). A similar study by Abriouel et al., profiled the biodiversity of the Spanish farmhouse cheese Alberquilla using PCR-TTGE. The 16S rRNA gene was amplified with results showing the presence of LABs such as *L. paracasei, L. brevis*, and *L. acidophilus* as well as less desirable species such as *E. coli* and enterococci (Abriouel et al., 2008).

It is noteworthy that TT/DGGE techniques can suffer from reproducibility-related issues due to variable staining, primer dimer formation and the loss of bands corresponding to less abundant strains in a community (Justé et al., 2008). Similar migration patterns of amplicons with similar melting domains but different sequences also pose a problem. Sekiguchi et al., found that a single DGGE band contained several different sequences (Sekiguchi et al., 2001). In addition, prior knowledge of the primer sequence is required for identifying a specific species or genus (Randazzo et al., 2009; Ndoye et al., 2011).

#### Single strand conformation polymorphism (SSCP)

SSCP allows for separation of DNA amplicons of similar size based on differences in the conformation of folded single strand DNA in a non-denaturing gel (Justé et al., 2008). Single strand nucleotide sequences fold into tertiary structures, depending on intramolecular interactions, under non-denaturing conditions and are then separated based on movement through an acrylamide gel (Duthoit et al., 2003; Jany and Barbier, 2008). This method was used by Takahashi et al., to study *hdc* genes in Gram-negative bacteria associated with Scombroid poisoning. Bands produced by SSCP were identified by comparison with reference strains and were successfully matched in 8 out of 10 fish samples (Takahashi et al., 2003). With respect to cheese, SSCP has not been extensively employed to assess defect-causing populations. Duthoit et al., used PCR-SSCP combined with microbial clone library sequencing (i.e., amplicons are cloned into vectors, and ultimately host cells, to facilitate DNA sequencing) to profile community dynamics of the raw milk Salers cheese during production. Universal and high GC primers were used to amplify regions of the 16S rRNA gene. Members of the LAB family including *L. lactis, S. thermophilus, L. plantarum*, and *E. faecium* were identified (Duthoit et al., 2003). SSCP has also been used to determine if certain cheese microbes can inhibit growth of *Listeria monocytogenes* by comparing communities in affected and unaffected cheeses. Saubusse et al., demonstrated that on day 8, cheese samples with the lowest counts of *L. monocytogenes* contained *Enterococcus faecium, Enterococcus saccharominimus, Chryseobacterium* spp., and *Corynebacterium flavescens, Lactococcus garvieae*, and *Lactococcus lactis*, respectively. Further studies revealed that *L. monocytogenes* inhibition occurred where *L. lactis, L. garvieae* and to a lesser extent *C. flavescens* and *E. saccharominimus* were present. This could be as a result of competitive inhibition or an indication of bacteriocin production (Saubusse et al., 2007).

#### Fluorescence in situ hybridisation (FISH)

FISH is based on hybridizing regions of a target bacterial genome to a taxon specific DNA probe labeled with a fluorescent dye. These regions can then be detected using fluorescence microscopy or flow cytometry (Justé et al., 2008). FISH requires prior knowledge of the microbial populations present in a sample (Randazzo et al., 2009). Ercolini et al., used FISH to detect *L. lactis, Lactobacillus plantarum*, and *Leuconostoc mesenteroides* in Stilton cheese. This approach was successfully used to identify microbes resident in different locations within the cheese matrix. *L. mesenteroides* colonies were found to be distributed throughout the cheese while *L. plantarum* was only found beneath the crust of the cheese. Lactococci were found in the core and veins (Ercolini et al., 2003). Bunthof et al., employed FISH and flow cytometry to study the viability of LABs using probes labeled with different dyes to discriminate between live and dead cells. The dyes were selected based on their spectroscopic properties to stain DNA. Carboxyfluorescein diacetate (cFDA), a non-fluorescent precursor which is converted to a fluorescent product by cellular enzymes, was used as a live cell stain. Impermeant exclusion dyes propidium iodide (PI) and cyanine dye TOTO-1 were attached to probes and used to stain dead cells. In experiments performed on bile salt stressed cultures of *L. lactis, L. helveticus*, and *L. mesenteroides* both TOTO-1 and cFDA proved to be accurate indicators of live and dead cells in comparison to plate counts (Bunthof et al., 2001).

#### Amplified ribosomal DNA restriction analysis (ARDRA) and terminal restriction fragment length polymorphism (T-RFLP)

ARDRA, also known as restriction fragment length polymorphism (RFLP), involves restriction enzyme digestion of multiple PCR amplicons (Justé et al., 2008). As restriction enzymes digest DNA at specific cleavage sites, differences in amplicon sequences may result in the absence or presence of cleavage sites. Gel electrophoresis of digested amplicons allows for comparative analyses. PCR products can be labeled, at the 5' and/or 3' ends, with a fluorescent dye and are then identified based on differences in multiple restriction enzyme sites (Justé et al., 2008; Randazzo et al., 2009). This method was used to study the microbial dynamics of the smear ripened Tilsit cheese by Rademaker et al., using conserved bacterial primers and two restriction enzymes (*Hae*III and *Cfo*I) (Rademaker et al., 2005). T-RFLP has been used for bacterial profiling in many dairy products however, these methods suffer from a lack of resolution and thus have been of limited use in complex food matrices (Justé et al., 2008).

#### Ribosomal intergenic spacer analysis (RISA)

RISA focuses on the 16S/23S ribosomal spacer region. The spacer region between these two genes represents a good target for bacterial identification due to heterogeneity in nucleotide length and sequence (Justé et al., 2008). RISA has been automated (automated RISA or ARISA) using fluorescently labeled PCR primers where a laser is used to detect fluorescent amplicons, (Justé et al., 2008). This method was used by Cardinale et al., to profile bacterial communities in goat's milk using universal primer sets. Results showed that the primer set employed is very effective for evaluating bacterial profiles in complex communities as it yields a wide range of spacer sizes (134–1387 bp), produces reproducible profiles and amplifies bacteria at DNA template concentrations from 280 to 0.14 ng/μl (Cardinale et al., 2004).

#### Denaturing high performance liquid chromotography (DHPLC)

DHPLC is a relatively new technique that has been employed to study microbial populations in the intestine and in environmental samples. This method involves the separation of PCR amplicons via an automated ion-pairing HPLC system (Jany and Barbier, 2008). Ercolini et al., used this technique in conjunction with DGGE to study natural whey cultures in Caciocavallo Silano cheese. PCR fragments generated after amplification of a region of 16S rRNA gene were separated by DHPLC on a C18 reverse phase column. Peaks generated by DHPLC were collected and sequenced. DHPLC generated the same results as DGGE, under the same conditions (Ercolini et al., 2008). Major advantages of this system include that it is fully automated and avoids gel preparation. However, problems with fragment co-migration or the presence of many copies of a DNA fragment may again result in inaccurate representation of microbial diversity (Ercolini et al., 2008).

#### DNA microarrays

DNA microarray technology, originally developed for gene expression analysis, has recently been adapted for profiling microbial communities (Zhou, 2003; Liu-Stratton et al., 2004). This approach is of particular interest because of its high density and high throughput capacity. DNA microarray technology is based on the hybridisation of fluorescently labeled target sequences to immobilized complementary sequences (oligonucleotides or small single strand PCR amplicons). The detector sequences are covalently attached to a solid support, either nylon or nitrocellulose membrane (low density macroarrays) or a glass slide (high density microarrays) (Bodrossy and Sessitsch, 2004; Liu-Stratton et al., 2004; Justé et al., 2008). Detector oligonucleotides are adapted to have nearly identical melting temperatures by including amine salts and/or by manipulating their lengths. The length of oligonucleotide probes are of key importance. Short probes of 20–25 nucleotides in length are preferred for microbial ecology studies and require PCR amplification of marker genes. Longer probes (50–70 nucleotides) yield better sensitivity and are therefore generally used for transcriptome studies. Long probes also do not require PCR amplification thus avoiding potential PCR bias issues (Bodrossy and Sessitsch, 2004). Target sequences, which are fluorescently labeled, then hybridize with complementary detector oligonucleotides to produce a detectable signal (Jany and Barbier, 2008; Justé et al., 2008).

There are three classes of microarrays, functional gene arrays (FGAs), community genome arrays (CGAs), and phylogenetic oligonucleotide arrays (POAs) (Zhou, 2003; Justé et al., 2008). FGAs are used to monitor the activity of genes that encode functional enzymes in microbial populations (Zhou and Thompson, 2002). CGAs consist of whole genomic DNA, isolated from pure cultures, which are used as a probe for profiling microbes in complex communities (Zhou and Thompson, 2002; Wu et al., 2004; Bae et al., 2005). CGA relies on fluorescence based detection on a non-porous surface and is of particular use for bacterial identification at the species and strain level (Zhou, 2003). A genome probing microarray (GPM) was used by Bae et al., to monitor community dynamics of LABs in the Korean fermented food Kimchi (Bae et al., 2005). The method employed could potentially be applied to a cheese matrix. The major disadvantage of CGAs is that only cultivable microbes in a community can be analysed because genomic DNA from pure isolates are required as probes (Zhou, 2003). POAs employ rRNA or other highly conserved sequences as phylogenetic probes. This approach allows for the analysis of both highly variable and highly conserved regions of bacterial DNA and can facilitate species level resolution. A 16S rRNA targeting microarray was used by Treimo et al., to quantify both *L. lactis* ssp. *lactis* as well as several species of propionibacteria in a liquid cheese model after 0 h, 24 h, 48 h, 7 days, and 5 weeks. DNA from the propionibacteria was shown to increase from 48 h up to 7 days, albeit at a slower growth rate than was observed in corresponding broth samples (Treimo et al., 2006). POAs were also used by Kostic et al., to identify pathogenic bacteria in a predominantly non-pathogenic community. Rather than using 16S rRNA, *gyrB* (encoding the B subunit of bacterial gyrase) was used as a phylogenetic marker in that instance.

There are some issues arising when attempting to apply DNA microarrays to analyse environmental or food samples. These fall into three main categories. Firstly, the diversity between the target and probe sequences, particularly in environmental samples, may affect hybridisation particularly if probes are sourced from pure cultures. Secondly, while recoverable DNA is not an issue when dealing with pure cultures, the amounts of DNA retrieved from environmental samples may be below accurate detection limits. Finally, the presence of hybridisation inhibitors in cheese may also be an issue (Zhou and Thompson, 2002).

## New detection methods: next generation sequencing (NGS)

Next generation, also known as massively parallel or high throughput, sequencing technologies represent a dramatic improvement over the traditional Sanger DNA sequencing method when it comes to investigating microbial communities (Morozova and Marra, 2008). High throughput screening can be applied to specific target genes, such as the 16S rRNA gene, as well as to (meta)genomic and (meta)transcriptomic applications (O'Flaherty and Klaenhammer, 2011). Sequencing of the 16S rRNA gene allows one to determine the relative proportions of different microbial populations within complex communities. In situations where there is a need to differentiate between species that are very closely related, and thus have highly conserved 16S rRNA genes, metagenomic sequencing, i.e., the analysis of the total genetic content of a particular community, is an alternative (Mardis, 2011; O'Flaherty and Klaenhammer, 2011). Whole genome sequencing of harmful (cheese defect bacteria) and beneficial bacteria (O'Flaherty and Klaenhammer, 2011) is also facilitated. Once entire genomes have been sequenced, comparisons can be made better to understand the relationships between microbes within a cheese matrix (O'Flaherty and Klaenhammer, 2011). The majority of NGS platforms currently employed are supplied by three companies i.e., Roche 454 (GS-FLX, GS-FLX+, GS Junior), Illumina (GA, GA II, HISEQ, MISEQ) and Applied Biosystems (ABI SOLiD). The data output for each of the above is summarized in Table 2 (Mardis, 2011; O'Flaherty and Klaenhammer, 2011). Less common systems include the Helicos Heliscope, Pacific Biosciences SMRT, Life Technologies Ion Torrent PGM and Oxford NanoPore Technologies (Mardis, 2008, 2011). NGS instruments share certain similarities such as the removal of the need for bacterial cloning. Sequences are typically amplified on a glass slide or within microbeads to produce sufficient signal for detection. NGS systems are also capable of sequencing DNA from both ends of single fragments or fragments which are many kbp apart. This process is termed paired end sequencing (Mardis, 2008, 2011).

**Table 2:** **List of Bases/Read and Yield/Run of the most common NGS platforms**.

**Instrument**	**Bases/Read**	**Yield (Mb)/Run**
Roche 454 GS Jr. Titanium	400	50
Roche 454 FLX Titanium	400	400
Roche 454 FLX+	650	650
Illumina MiSeq	150 + 150 (Paired end)	1200
Illumina GAII	150 + 150 (Paired end)	96000
Illumina HiSeq 2500	150 + 150 (Paired end)	180000
Illumina HiSeq 2000	100 + 100 (Paired end)	600000
SOLiD 5500 × l	75 + 35	155100

### Roche 454 FLX pyrosequencer

The Roche 454 pyrosequencing based technology was first released in 2005 (Morozova and Marra, 2008) and relies on the generation of a library of DNA fragments which are hybridized to beads. These beads carry oligonucleotide sequences that complement adaptor sequences ligated to the DNA fragments of interest (Mardis, 2008). The bead/fragment complex is then amplified using emulsion PCR in an aqueous microreactor (Roh et al., 2010). After emulsion PCR, amplification fragments are sequenced in a picotiter plate. Within the picotiter plate, a sequencing-by-synthesis approach is used to measure the release of pyrophosphate (PPi). The response to the incorporation of a complementary nucleotide is then measure by a Charge-Coupled Device (CCD) (Morozova and Marra, 2008).

The use of this and other NGS based technologies allows for the study of microbial populations in many environments including foods, and is of particular use in examining spatial and/or temporal variability of a specific microbial community (Roh et al., 2010). Indeed, this technology has been used by Quigley et al., to investigate the sub-dominant bacteria in artisanal cheeses. More specifically, 116,000 16S rRNA amplicon reads, corresponding to 62 different cheese types, were sequenced to reveal the presence of five bacterial phyla including *Firmicutes, Bacteriodetes, Proteobacteria, Acintobacteria* and the fungal phylum *Ascomycota*. Indeed, several genera not previously associated with cheese, including *Faecalibacterium, Prevotella*, and *Helcococcus* were detected and, for the first time, the presence of *Arthrobacter* and *Brachybacterium* in goats' milk cheese was noted. The detection of populations not previously associated with cheese shows the benefits of using high throughput screening to investigate these microbial populations (Quigley et al., 2012). Masoud et al., have also used this technology to profile the microbial communities present in Danish raw milk and cheeses at different stages of ripening. This study showed that the microbial diversity of Danish raw milk cheeses declined during ripening. This is due to the impact of the cooking temperature and acidification that occur prior to and during the ripening process. Further studies into the effects of cooking temperature, acidification and starter culture addition on the growth of pathogenic bacteria including *E. coli, Listeria innocua*, and *S*. *aureus*, in four inoculated cheeses, was investigated using both NGS and qPCR. Results showed that *E. coli* numbers increased until day 7 of ripening and then decreased thereafter. Adjunct starters *Brevibacteria linens* and *Microbacterium lacticum* also did not affect growth of the pathogenic strains during ripening (Masoud et al., 2011). Roche-based pyrosequencing was also used by Alegria et al., to investigate the microbial biodiversity within the traditional Polish cheese Oscypek. Four bacterial phyla were identified i.e., *Firmicutes, Actinobacteria, Bacteriodetes*, and *Proteobacteria*. This was also the first observation of *Bifidobacteriaceae* present in cheese as sub-dominant populations belonging to both *Bifidobacteriaceae* and *Moraxellaceae* were identified using pyrosequencing (Alegria et al., 2012).

The 454 pyrosequencing can also be used to sequence the genomes of many dairy associated bacteria. This would allow for determining particular species which contain a desirable gene cluster, such as biogenic amine gene clusters. Examples of cheese associated microbes sequenced include, *Lactobacillus cypricasei* KCTC 13900 (Kim et al., 2011), *Corynebacterium casei* UCMA 3821 (Monnet et al., 2012), *Streptococcus macedonicus* ACA-DC 198 (Papadimitriou et al., 2012), and *Corynebacterium variabile* DSM 44702 (Schroder et al., 2011) among many others.

### Illumina/solexa genome analyzer

The Illumina Genome Analyzer was commercially released in 2006 (Zhang et al., 2011) and has since been updated in the form of the HiSeq and MiSeq platforms. For these instruments single stranded DNA fragments are attached to a flow cell, a solid, multi-channel single molecule array (Mardis, 2008). DNA fragments are attached to the flow cell via an adaptor molecule and form bridges by hybridizing to complementary adaptors. The bridge is then used as the template for generation of complementary strands through bridge amplification (Morozova and Marra, 2008). After amplification, the flow cell contains upwards of 40 million clusters, where each cluster contains clones of the template DNA fragment (Morozova and Marra, 2008). This system also uses sequencing by synthesis approach except that all four nucleotides are added together with a DNA polymerase rather than individually as in the 454 system. The DNA polymerase incorporates fluorescently labeled reversible terminator sequences to growing nucleotide chains. Each terminator sequence is labeled with a different fluorophore to differentiate between the different nucleotide bases. Therefore, each cluster is sequenced by the color associated with the nucleotide added (Mardis, 2008; Morozova and Marra, 2008; Zhang et al., 2011). It has recently been established that Illumina based 16S rRNA sequencing is a valid alternative to other 16S based sequencing approaches (Lazarevic et al., 2009).

### ABI SOLiD

The Applied Biosystems SOLiD sequencer was released in 2007 and relies on sequencing by ligation rather than by synthesis (Morozova and Marra, 2008). Sequencing libraries are generated by emulsion PCR similar to 454 sequencing and amplified products are then sequenced on a glass surface by repeating rounds of hybridisation and ligation with 8-mer fluorescent oligonucleotides. The 8-mer oligonucleotides contain fluorescent markers that identify a two base combination which is termed di-nucleotide encoding (Zhang et al., 2011). The 2 base encoding method allows for an accuracy of 99.94%. The library preparation however, is time consuming (Morozova and Marra, 2008). To date this system has not been used to investigate cheese microbiology.

It is anticipated that these and new sequencing technologies will be widely employed to provide a detailed insight into cheese-associated microbial populations in the future.

## Conclusions and future perspectives

Traditional culture-based approaches to detect bacteria in cheeses are being replaced by culture-independent molecular methods. Researchers are shifting from a polyphasic approach which relies on both culture-dependent and independent techniques to PCR based culture-independent methods only. This is due to the rapid ability of PCR to detect viable, non-viable, damaged/permeabilized and non-cultivable microbes. Molecular methods, therefore, allow for more effective studies of dominant and sub-dominant populations in complex matrices such as cheese, promoting a greater understanding of microbial community structure and activity. The relationships between different microbes as well as the different pathways involved in creating many of the varieties of cheese are now better understood than ever before (Justé et al., 2008; Ndoye et al., 2011; Quigley et al., 2011).

While the advent of PCR has revolutionized the way in which microbes are detected in food products, it is important to note that there is no “one size fits all” PCR-based approach. Thus, selecting the correct method/s for sample analysis is as important as the technique itself. Techniques such as PCR-TTGE/DGGE and SSCP provide some insight to microbes present in a food sample and have thus predominantly been used for population based studies. Conventional PCR or qPCR are more frequently employed when targeting specific taxa or genes. DNA microarrays can also be employed in a number of situations, depending on which genes are present on the array. qPCR based approaches are already available to detect and quantify decarboxylase gene expression in fermented foods. In the case of decarboxylase genes, sequence variability has led to the development of multiplex PCR assays to facilitate the simultaneous detection of the major enzyme groups (Coton and Coton, 2005; Fernández et al., 2006; Torriani et al., 2008; Ladero et al., 2012). Notably, current BA detection is often through HPLC, with a detection limit of 0.1 mg/kg. However, this does not assist in pre-empting product recall issues. Thus quantifying the levels of certain decarboxylase genes present via qPCR or DNA microarrays, at various stages of production, could potentially prevent contaminated products entering the market and consequently reduce product recall costs.

Finally, next generation sequencing represents the most recent advance with respect to the evolution of microbial ecology. NGS will significantly enhance our understanding of the genomes and transcriptomes of food microbes and provide greater insight into structural community interactions and metabolic activity. Further reductions in labor time and costs will make NGS even more attractive for food quality and safety studies (Quigley et al., 2011).

### Conflict of interest statement

The authors declare that the research was conducted in the absence of any commercial or financial relationships that could be construed as a potential conflict of interest.
